# Aesthetic sensitivity: relationship with openness to experience and agreeableness, health-related quality of life and adaptive coping strategies in people with high sensory processing sensitivity

**DOI:** 10.3389/fpsyg.2023.1276124

**Published:** 2024-01-10

**Authors:** Antonio Chacón, Mercedes Borda-Mas, Francisco Rivera, Manuela Pérez-Chacón, María Luisa Avargues-Navarro

**Affiliations:** ^1^Spanish Association of Highly Sensitive Professionals and Psychologists, PAS España, Madrid, Spain; ^2^Department of Personality, Assessment, and Psychological Treatment, University of Seville, Seville, Spain; ^3^Department of Experimental Psychology, University of Seville, Seville, Spain

**Keywords:** highly sensitive person, general health, mental health, emotional role, vantage sensitivity, creativity, empathy

## Abstract

Aesthetic sensitivity in people with high sensory processing sensitivity (SPS) reflects the positive perception of life, especially aspects related to the arts and nature. This study is focused on the analysis of the effect of aesthetic sensitivity in relation to indicators of health-related quality of life (general health, mental health and emotional role), the personality traits openness to experience and agreeableness, and coping strategies in people with SPS. The adult participants (*N* = 10,520, mean age = 33.61) completed the Spanish versions of the High Sensitivity Person Scale (HSPS-S), Short Form Health Survey (SF-36), NEO Five Factor Inventory (NEO-FFI) and Coping Strategies Inventory (CSI). It was observed that people with high aesthetic sensitivity presented greater openness and agreeableness, tended to use adaptive coping strategies and showed a slightly poorer functioning in different areas of daily living. Moreover, health-related quality of life, mental health and adaptive coping strategies occupied central positions in the correlations between variables, with a positive impact between mental health and adaptive coping strategies with openness and agreeableness. Lastly, the level of aesthetic sensitivity did not play a moderator role, and it exerted no differential influence on its relationship with the analysed variables. Now, it has been found that people with high levels of aesthetic sensitivity cope more adequately, which would cushion the effect that high SPS can have on mental health, specifically on anxious and depressive symptoms. It is concluded that these findings are relevant and useful for future propositions of prevention and clinical intervention, as well as for counselling in the psychoeducational, labour and family scopes, amongst others.

## 1 Introduction

Sensory Processing Sensitivity (SPS) is a non-pathological personality trait that is present in 25–30% of the general population (Lionetti et al., 2018; Pluess et al., 2018). People with high SPS are characterised by perceiving subtle details of the environment, deeper processing of small perceived stimuli, and a tendency to feeling overwhelmed more easily in very stimulating environments (Aron et al., 2012).

Previous studies on psychological variables and SPS have focused on identifying risk factors associated with high levels of this trait, as well as its negative consequences. However, little research studying certain personality traits and coping strategies that activate specific areas of brain activity (Acevedo et al., 2017) highlights positive qualities of SPS. Similarly, very few studies have delved into the extent to which this positive facet in people with high SPS would be related to better health-related quality of life. In this sense, studies based on network analysis allow determining how the variables are grouped, as well as the importance of each the variables within the network, showing strong or weak connections depending on the position they occupy in it (central or peripheral). Analysing the positive facet in relation to certain personality traits, coping strategies and health indicators from a network analysis model offers a novel perspective in the understanding of high SPS.

Initially, following the diathesis-stress model, this trait was associated with vulnerability and worse adaptability to stressful environments in people with high sensitivity (Ellis and Boyce, 2011). However, from the theory of vantage sensitivity, there are also individual differences in the responses to positive stimuli (Pluess and Boniwell, 2015; Lionetti et al., 2018; Villiers et al., 2018). Furthermore, from the theory of differential susceptibility (Belsky and Pluess, 2009; Pluess and Belsky, 2009), it is stated that highly sensitive people present greater (either positive or negative) effects, that is, greater emotional reactivity in favourable or unfavourable environments, respectively.

The central nervous system in SPS acquires, selects and processes sensory information in a particular manner. In addition to greater sensitivity to the exposure to negative stimuli, there is also a better use of the positive aspects of situations and interactions (Aron et al., 2012). From Gray’s Reinforcement Sensitivity Theory (RST) (Gray, 1982), high sensitivity is predominated by the control-pause system and the tendency to stop before taking action (Aron, 2017), adopting different coping strategies depending on the environmental challenges. Thus, the activation of the Behavioural Inhibition System (BIS) is associated with the tendency towards avoidance and social withdrawal, as a consequence of the overstimulation caused especially by the stimuli of social interaction (Pérez-Chacón et al., 2023a,b). On the other hand, the activation of the Behavioural Activation System (BAS) in highly sensitive people is associated with empathy (Pérez-Chacón et al., 2021), creativity and a high degree of integrity (Aron, 2017), and attraction to beauty in the arts and nature, which are strengths that contribute to emotional well-being. Empathy and the creativity derived from the greater sensitivity to subtleties and a deeper processing of information grant these people the ability to identify and solve relational problems (Aron et al., 2019), showing a constant willingness to help and support other people.

These positive traits in highly sensitive people are related to aesthetic sensitivity, which is characterised for the capacity to capture subtleties and perceive the world globally (i.e., from different points of view), to intuit beyond words, to enjoy, to feel any aspect of life in a positive manner (especially those related to the arts and nature), to delve into multiple topics, and to be interested in the meaning of life, helping people, animals and the environment. In this sense, aesthetic sensitivity is related to positive affection (Sobocko and Zelenski, 2015) and the activation of brain areas related to reward and empathy (Acevedo et al., 2017).

Therefore, several authors refer to the relationship of SPS with certain personality traits, such as agreeableness and openness, with cognitive exploration, a refined sense of aesthetics, emotional involvement and creative fantasy being key elements of these traits (DeYoung, 2015). On the other hand, sensitivity oriented to the aesthetics of the environment and sensitivity to the social world (i.e., perceiving socioemotional actions and attempting to regulate the emotional distress of other people) (Trå et al., 2022) constitute a pattern that coincides with the characteristics of agreeableness (harmonious, cooperative people who tend to withdraw from social conflicts and know how to correctly read the minds of other people) (Nettle and Liddle, 2008) and openness to experience (curious, imaginative and flexible people who consider new ideas, behaviours and feelings) (McCrae and Costa, 1997). Thus, these personality traits are expected to be frequently present in people with high sensitivity.

In this sense, previous studies conducted with the High Sensitivity Person Scale (HSPS) (Aron and Aron, 1997) show that aesthetic sensitivity (AES) is related to the two personality traits mentioned (Smolewska et al., 2006; Lionetti et al., 2019; Trå et al., 2022). Regarding openness to experience, the literature reports a positive relationship with high sensitivity (Smolewska et al., 2006; Lionetti et al., 2018). On the other hand, in regard to agreeableness, the findings are less conclusive, with Trå et al. (2022) observing a relationship with high sensitivity. However, this relationship was weak in the study of Lionetti et al. (2019).

In terms of health and its relationship with coping style, the SPS could be acting by moderating the impact that the use of certain coping strategies can have on health. Although, in general, strategies focused on the problem are associated with better health, and those focused on emotion are associated with worse physical and mental health (Zimmer-Gembeck and Skinner, 2015), in people with high sensitivity, coping skills focused on emotion are negatively related to depressive tendencies (Yano et al., 2021). Moreover, they are also associated with different physical health problems, such as pain, which could be due to a greater perception of physical and internal signs (Benham, 2006), as well as to psychopathology, such as anxiety and depression (Liss et al., 2008). In turn, positive traits such as empathy improve the results in emotional and mental quality of life (Genizi et al., 2019) by focusing help on the well-being of other people, whilst creativity, interest and open-mindedness favour vitality.

Furthermore, in terms of quality of life, previous studies have detected low life and job satisfaction in hypersensitive people (Sobocko and Zelenski, 2015). Similarly, overstimulation and low sensory threshold are associated with lower levels of life satisfaction and negative affection (Booth et al., 2015; Sobocko and Zelenski, 2015), especially in those people with greater life stress and worse emotional regulation (Brindle et al., 2015). In this way, and in line with vantage sensitivity, the relationship between SPS and life satisfaction is moderated by the environmental conditions and the specific facet of the trait (Jagiellowicz et al., 2020). In addition, from positive psychology (Seligman and Csikszentmihalyi, 2003), different authors have studied strengths such as creativity, openness, appreciation of beauty, and vitality, amongst others, coinciding with the positive side in people with high SPS. Developing these characteristics or strengths leads to positive experiences, states of well-being, satisfaction with life and, ultimately, better quality of life.

To sum up, despite the fact that SPS is a trait that has been studied more frequently in the population in the last years, there are very few conclusive studies on the facet of aesthetic sensitivity in relation to studies on openness, agreeableness, health-related quality of life (physical health, mental health and emotional role or degree to which emotional problems interfere with work or other daily activities) and coping strategies (active/non-active adaptive and non-adaptive). Therefore, this study analysed the possible effect of aesthetic sensitivity as a positive characteristic of highly sensitive people and its relationship with openness and agreeableness, the variables of health-related quality of life, and coping strategies.

From all of the above, three objectives were set for this study: 1) analyse the behaviour of the different variables (indicators of health-related quality of life, openness and agreeableness, and coping strategies) as a function of the level of aesthetic sensitivity (low, medium and high); 2) verify the relationships between pairs of variables and determine whether these relationships are moderated by the level of aesthetic sensitivity; and 3) explore, through network analysis, the relevance of each variable in the set of relationships between all variables and determine whether there are modifications due to the moderator effect of aesthetic sensitivity in the set of relationships obtained.

Based on the objectives set for this study, it was expected that, in view of the variability of positive experiences, aesthetic sensitivity could be associated with health-related quality of life, the use of adaptive coping strategies and the personality traits openness and agreeableness. Moreover, it was also expected that the presence of high aesthetic sensitivity would contribute to a greater association between the analysed variables, acting as a moderator. Finally, it is expected that, in the general relationship between the variables, indicators of quality of life related to health and the personality traits openness and agreeableness will occupy a central position, with coping strategies appearing in a peripheral position. In addition, the level of aesthetic sensitivity is expected to act as a moderator in the general relationship between the variables.

Determining whether the relationships between health-related quality of life, coping strategies and openness to experiences and agreeableness are moderated by the level of aesthetic sensitivity would be useful for helping to counteract the emotional reactivity to negative stimuli and managing the characteristics of SPS satisfactorily and efficiently in highly sensitive people.

## 2 Materials and methods

### 2.1 Participants

The sample consisted of 10,520 adults (1739 men and 8,781 women) (mean age 33.61 years, SD = 11.39; range 18–79 years). The participants were recruited in the community context of Spain, by convenience sampling and by sample accessibility.

They all met the inclusion criteria: a) being at least 18 years old; b) providing all the data and completing the battery of tests; and c) signing the informed consent. The characteristics of the participants are presented in Table 1.

**Table 1:** Characteristics of the participants (N = 10,520).

	Men (n = 1,739)		Women (n = 8,781)	
	n	%	n	%
*Age*				
Mean (Range: 18–79)	34.60		33.41	
SD	12.34		11.19	
				
*Age group*				
≤ 30	786	7.47	4,030	38.31
31–40	436	4.14	2,392	22.73
41–50	303	2.88	1,649	15.67
51–60	159	1.51	587	5.58
≥ 61	55	0.52	123	1.17
*Marital status*				
Single	996	9.47	4,262	40.51
With partner	245	2.33	1,674	15.91
Married	303	2.88	1,826	17.36
Divorced	140	1.33	712	6.77
Widowed	4	0.04	38	0.36
Not specified	51	0.48	269	2.56
*Education level*				
College	893	8.49	5,314	50.51
High school	640	6.08	2,827	26.87
Secondary	150	1.43	529	5.03
Primary	50	0.47	101	0.96
Without studies	6	0.06	10	0.09

### 2.2 Procedure

This is a prospective, cross-sectional, survey-based study. It was conducted by faculty members with renowned research experience and experts in the knowledge of people with high sensitivity, experienced specialists and managers in the PAS Spain association. These professionals led the process of dissemination and access to the online link for participation in this investigation (see Pérez-Chacón et al. (2023a,b) and Chacón et al. (2023) for further information about the process).

This study followed the code of ethics of the World Medical Association (2013), and it was approved by the University where it was developed.

The data were gathered using an anonymous survey, excluding any data that could allow identifying the participants. The survey was accessed through the *Microsoft Form* platform, which was distributed through the website of the PAS Spain association and social media. Upon entering the platform, after providing the link, information was made available about the relevant characteristics of the study (objectives, the absence of possible risks and rewards of participating, the necessary time to complete it, etc.), as well as other complementary information (e.g., the possibility of taking breaks). The participants were informed of their right to leave the study whenever they wished to, and to access and cancel their data, in compliance with Organic Law 3/2018, of December 5^th^, on the Protection of Personal Data and Guarantee of Digital Rights. Furthermore, the contact information of the researchers was made available to the participants. Lastly, the participants were allowed to state, by ticking the box designed for this purpose, whether they agreed to participate or not, as well as their consent to initiate and proceed to their participation in the terms presented in the screen corresponding to the informative Sheet. The participants could only access the survey if they had previously marked the agreement option. The battery of tests used in this study did not include sensitive information or information that could indicate psychopathology. However, upon completing the survey, the participants were given the option of introducing a unique four-digit code that was only known to each participant, in case they wished to exercise their right to access and cancel their data or revoke their consent.

### 2.3 Data analysis

For the first objective, the sample was divided into three groups as a function of aesthetic sensitivity (low, medium and high) from percentiles 33 (low level = score ≤ 35) and 66 (high level = score ≥ 38). In each level, the mean scores and standard deviations were calculated for the indicators of health-related quality of life, personality traits (openness and agreeableness) and coping strategies. Then, mean difference analyses were performed through single-factor ANOVA, and the effect size was estimated using eta squared, with the following values: no effect (η^2^ = <0.010), small effect (η^2^ = 0.010–0.059), moderate effect (η^2^ = 0.060–0.140) and large effect (η^2^= > 0.140) (Lenhard and Lenhard, 2016).

In the second objective, Spearman’s correlation coefficient was used, interpreting its significance and the value of the correlation as effect size, with the following values: no effect (*r*_sp_ = <0.10), small effect (*r*_sp_ = 0.10–0.30), moderate effect (*r*_sp_ = 0.31–0.50) and large effect (*r*_sp_= > 0.50). Furthermore, the moderator effect of aesthetic sensitivity on the bivariate correlations of the analysed variables was assessed by calculating the segmented correlations in the three levels of aesthetic sensitivity. Subsequently, Cohen’s *q* was applied as estimator of the effect size of the differences of the correlations between the levels of aesthetic sensitivity, with the following values: no effect (*q =* <0.1), small effect (*q = 0*.10–0.30), moderate effect (*q = 0*.31–0.50) and large effect (*q*= > 0.50).

Finally, for the third objective, a network analysis of all variables was carried out, estimating the weights of the matrix using the EBICglasso Networks method (Foygel and Drton, 2010). Then, an additional network analysis was performed, following the same estimation method, segmenting the sample as a function of the level of aesthetic sensitivity to observe the changes in the parameters of the network analysis and determine the possible moderator effect.

All the data analyses were carried out using JASP software v0.17.2 (JASP Team, 2023). In addition, the calculation instruments provided by Psychometrica were employed for the second objective (Lenhard and Lenhard, 2016). In the different sections of the results, to control for the potential confounding effects of sex and age, a control method was implemented by calculating standardised residuals for the study variables. This approach involved adjusting the raw scores of the variables for sex and age, resulting in residuals that represent the deviation of each individual’s score from the expected score based on their sex and age. These standardised residuals were then used in subsequent data analyses. This method ensures that the analyses of the relationships between aesthetic sensitivity, health-related quality of life, personality traits, and coping strategies are not unduly influenced by these demographic factors, thereby enhancing the accuracy and validity of the findings (Tabachnick et al., 2013).

### 2.4 Measures

The Spanish adaptations were used, as well as several subscales of the following instruments, indicating their internal consistency with alpha (α) and McDonald’s Omega coefficients (ω): 1) High Sensitivity Person Scale (HSPS-S) (Chacón et al., 2021), to identify people with high sensitivity, with subscale *aesthetic sensibility* (AES: awareness of the aesthetics of the environment) (α and ω = 0.79); 2) Short Form-36 Health Survey (SF-36) (Vilagut et al., 2005), to evaluate health-related quality of life, with subscales *general health* (GH: personal valuation of health that includes mental health, perspectives of health in the future, and resistance to falling ill) (α = 0.84 and ω = 0.83), *mental health* (MH: feelings of happiness, calmness, and tranquility vs. feelings of anxiety and depression) (α and ω = 0.87), and *emotional role* (ER: functioning in different domains of daily life due to emotional problems) (α and ω = 0.82); 3) Personality Inventory NEO-FFI (Cordero et al., 1999), with subscales *openness to experience* (O: search for and active valuation of experience) (α = 0.79 and ω = 0.80), and *agreeableness* (A: evaluates interpersonal tendencies) (α = 0.76 and ω = 0.78); and 4) Coping Strategies Inventory (CSI) (Cano-García et al., 2007), with adaptive coping strategies (ACS) and maladaptive coping strategies (MCS). ACS included *problem solving* (PS) (modifying the situation) (α and ω = 0.82), *cognitive restructuring* (CR) (modifying the meaning of the situation) (α = 0.74 and ω = 0.77), *social support* (SS) (searching for emotional support) (α and ω = 0.86) and *emotional expression* (EE) (freeing one’s emotions) (α = 0.82 and ω = 0.84). On the other hand, MCS included *active* MCS (A_MCS) and non-active MCS (Na_MCS). A_MCS included *wishful thinking* (wishing that reality were different) (α = 0.86 and ω = 0.87) and *self-criticism* (blaming oneself for the situation) (α and ω = 0.88), whereas Na_MCS included *problem avoidance* (avoiding and withdrawing from actions or thoughts) (α and ω = 0.70) and *social withdrawal* (avoiding and withdrawing from people) (α = 0.77 and ω = 0.79). A global indicator was used with the four adaptive strategies (PS + CR + SS + EE) (α and ω = 0.88), as well as for A_MCS (α and ω = 0.90) and Na_MCS (α = 0.74 and ω = 0.73) (see Pérez-Chacón et al., 2023a,b for further information about the instruments).

## 3 Results

With regard to the first objective, i.e., to analyse the behaviour of the different variables (indicators of health-related quality of life, openness and agreeableness, and coping strategies) as a function of the level of aesthetic sensitivity (low, medium and high), significant differences were observed in emotional role (M = 58.01, M = 52.90, M = 50.01, respectively), with small effect size (*η^2^* = 0.012) (Table 2).

**Table 2:** Means (standard deviations), significance test and effect sizes of aesthetic sensitivity levels with the different variables.

Descriptive statistics				Significance test and effect size			
Aesthetic sensitivity (HSPS-S) (range: 6–42)							
	Low (P_33_ = ≤35)	Medium	High (P_66_ = ≥38)				
	M(SD)	M(SD)	M(SD)	*F*	*p*	*η^2^*	
Health-related Quality of Life (SF-36) (range: 0–100)							
General health	62.41 (20.63)	61.10 (20.85)	60.57 (22.22)	8.95	***	0.002	
Mental health	50.76 (16.76)	49.79 (16.43)	49.13 (16.88)	24.64	***	0.005	
Emotional role	58.01 (30.63)	52.90 (31.11)	50.01 (32.48)	126.56	***	0.024	(s)
Personality traits (NEO FFI) (range: 0–60)							
Openness (P_50_ = 28)	29.93 (5.79)	33.98 (5.34)	37.13 (5.34)	1736.77	***	0.248	(l)
Agreeableness (P_50_ = 34)	29.14 (5.64)	30.34 (5.61)	31.75 (6.06)	142.76	***	0.026	(s)
Coping Strategies (CSI) (range: 0–40)							
Adaptive	10.26 (3.07)	11.11 (3.05)	11.97 (3.21)	292.93	***	0.053	(s)
Active maladaptive	12.36 (4.42)	12.72 (4.40)	13.16 (4.51)	53.82	***	0.009	
Non-active maladaptive	7.25 (3.11)	7.07 (3.04)	7.18 (3.19)	4.08		0.001	

***p < 0.001.

The descriptive statistics include, in the following order, the mean and standard deviation (in brackets).

Adaptive coping strategies: problem solving + cognitive restructuring + social support + emotional expression; Active maladaptive coping strategies: self-criticism + wishful thinking; Non-active maladaptive coping strategies: problem avoidance + social withdrawal. P50 = Percentile 50, P33 = Percentile 33, P66 = Percentile 66.

High scores in SF-36, NEO = -FFI and CSI indicate greater presence of the different variables: better quality of life, greater openness and agreeableness and greater tendency to use coping strategies. The levels of interpretation of η2 are: (s) small (η2 = 0.010–0.059), (m) moderate (η2 = 0.060–0.140) and (l) large (η2= > 0.140).

Regarding the personality traits, i.e., openness and agreeableness, significant differences were observed as a function of low (M = 29.93), medium (M = 33.98) and high aesthetic sensitivity (M = 37.13), as well as in agreeableness (M = 29.13, M = 30.34 and M = 31.75, respectively), with large effect size (*η^2^* = 0.236) in openness and small effect size (*η^2^* = 0.036) in agreeableness.

With respect to coping strategies, the adaptive strategies showed significant differences between the groups with low (M = 10.26), medium (M = 11.11) and high (M = 11.97) aesthetic sensitivity, with small effect size (*η^2^* = 0.051). The participants with high aesthetic sensitivity tended to use problem solving, cognitive restructuring, social support and emotional expression more frequently than the participants with low aesthetic sensitivity.

In the second objective, we verified, on the one hand, the relationships between pairs of variables and, on the other hand, we determined whether these relationships were moderated by the level of aesthetic sensitivity. In relation to the indicators of health-related quality of life (general health, mental health and emotional role), positive and statistically significant relationships were detected between mental health and emotional role, with large effect size (*r*_sp_ = 0.58) and for general health and mental health with emotional role, with medium effect size (*r*_sp_ = 0.45 and *r*_sp_ = 0.36, respectively) (Table 3).

**Table 3:** Relationships between the indicators of health-related quality of life, openness and agreeableness and coping strategies.

	(1)	(2)	(3)	(4)	(5)	(6)	(7)
Mental Health^(2)^	0.45*** (m)							
Emotional Role^(3)^	0.36*** (m)	0.58*** (l)						
Openness^(4)^	−0.02*	−0.06***	−0.18*** (s)					
Agreeableness^(5)^	0.10*** (s)	0.17*** (s)	0.04***	0.21*** (s)				
Adaptive Coping Strategies^(6)^	0.19*** (s)	0.26*** (m)	0.08***	0.26*** (s)	0.20*** (s)			
Active maladaptive coping strategies^(7)^	−0.27*** (s)	−0.50*** (l)	−0.37*** (m)	0.10*** (s)	−0.06***	−0.05***		
Non-active maladaptive coping strategies^(8)^	−0.08***	−0.15*** (s)	−0.13*** (s)	0.01	−0.09***	−0.22*** (s)	0.26*** (s)	

* *p* < 0.05 *** *p* < 0.001.

(1) = General health, (2) = Mental health, (3) = Emotional role, (4) = Openness, (5) = Agreeableness, (6) = Adaptive coping strategies, (7) = Active maladaptive coping strategies, (8) = Non-active maladaptive coping strategies.

The value of Spearman’s Rho (rsp) is included. The levels of interpretation of Cohen’s q are as follows: (s) = small (q = 0.10–0.30), (m) = moderate (q = 0.31–0.50) and (l) = large (q= > 0.50).

Furthermore, positive relationships were also observed between the two personality traits, i.e., openness and agreeableness, in a more peripheral position (*r*_sp_ = 0.21), between openness and mental health, through adaptive coping strategies (A-ACS *r*_sp_ = 0.26 and ACS-MH *r*_sp_ = 0.26, respectively) and between agreeableness and mental health (*r*_sp_ = 0.17), as well as through adaptive coping strategies (*r*_sp_ = 0.19). There was also a positive and statistically significant relationship between active and non-active maladaptive coping strategies (*r*_sp_ = 0.26). The effect size in all relationships was small.

On the other hand, negative and statistically significant relationships were identified in the indicators of health-related quality of life, for mental health and emotional role with active maladaptive coping strategies, with large (*r*_sp_ = −0.50) and medium effect size (*r*_sp_ = −0.37). There was also a negative and statistically significant relationship between adaptive coping strategies and non-active coping strategies (*r*_sp_ = −0.22), with small effect size. The other relationships were either statistically significant with negligible effect sizes or non-significant.

Subsequently, to determine whether these relationships were moderated by the level of aesthetic sensitivity, bivariate correlations were performed as a function of the level of aesthetic sensitivity: low, medium and high. Then, we determined the difference of correlations between the three levels of aesthetic sensitivity in the study variables, through the value of Cohen’s *q* (Table 4).

**Table 4:** Analysis of the moderator effect of aesthetic sensitivity level on the relationships between the indicators of health-related quality of life, openness, agreeableness and coping strategies.

		Aesthetic sensitivity level Low^l^/Medium^m^/High^h^						(1)	(2)	(3)	(4)	(5)	(6)	(7)
Mental Health^(2)^	0.46/0.45/0.44 ^l-m^*q = 0*.01 ^l-h^*q = 0*.01 ^m-h^*q = 0*.03						
Emotional Role^(3)^	0.38/0.33/0.36 ^l-m^*q* ^l-m^ *= 0*.04 ^m-h^*q = 0*.02 ^l-h^*q = 0*.03	0.60/0.57/0.57 ^l-m^*q = 0*.04 ^m-h^*q* < 0.01 ^l-h^*q = 0*.06					
Openness^(4)^	−0.01/0.03/0.004 ^l-m^*q* ^l-m^ *= 0*.01 ^m-h^*q = 0*.02 ^l-h^*q = 0*.01	−0.06/−0.02/−0.01 ^l-m^*q = 0*.04 ^m-h^*q = 0*.01 ^l-h^*q = 0*.04	−0.14/−0.10/−0.11 ^l-m^*q = 0*.04 ^m-h^*q = 0*.01 ^l-h^*q = 0*.03				
Agreeableness^(5)^	0.12/0.12/0.09 ^l-ml-m^*q = 0*.01 ^m-h^*q = 0*.04 ^l-h^*q = 0*.04	0.18/0.21/0.17 ^l-m^*q = 0*.03 ^m-h^*q = 0*.04 ^l-h^*q = 0*.01	0.06/0.09/0.4 ^l-m^*q = 0*.03 ^m-h^*q = 0*.02 ^l-h^*q = 0*.03	0.15/0.14/0.17 ^l-m^*q = 0*.01 ^m-h^*q = 0*.03 ^l-h^*q = 0*.02			
Adaptive coping strategies^(6)^	0.21/0.17/0.21 ^l-m^*q = 0*.03 ^m-h^*q = 0*.04 ^l-h^*q* < 0.01	0.26/0.25/0.31 ^l-m^*q = 0*.01 ^m-h^*q = 0*.06 ^l-h^*q = 0*.05	0.10/0.11/0.13 ^l-m^*q = 0*.01 ^m-h^*q = 0*.02 ^l-h^*q = 0*.03	0.18/0.17/0.18 ^l-m^*q < 0*.01 ^m-h^*q < 0.*01 ^l-h^*q < 0*.01	0.18/0.16/0.17 ^l-m^*q = 0.*02 ^m-h^*q = 0*.01 ^l-h^*q = 0*.01		
Active maladaptive coping strategies^(7)^	−0.27/−0.29/−0.26 ^l-m^*q = 0*.02 ^m-h^*q = 0*.03 ^l-h^*q = 0*.01	−0.50/−0.49/−0.49 ^l-m^*q = 0*.01 ^m-h^*q < 0.*01 ^l-h^*q = 0*.01	−0.39/−0.36/−0.32 ^l-m^*q = 0*.02 ^m-h^*q = 0*.04 ^l-h^*q = 0*.06	0.09/0.03/0.05 ^l-m^*q = 0*.05 ^m-h^*q = 0*.02 ^l-h^*q = 0*.03	−0.05/−0.11/−0.09 ^l-m^*q = 0*.05 ^m-h^*q = 0*.02 ^l-h^*q = 0*.04	−0.06/−0.08/−0.10 ^l-m^*q = 0*.02 ^m-h^*q = 0*.02 ^l-h^*q = 0*.04	
Non-active maladaptive coping strategies^(8)^	−0.09/−0.05/−0.08 ^l-m^*q = 0*.03 ^m-h^*q = 0*.03 ^l-h^*q = 0*.01	−0.14/−0.11/−0.14 ^l-m^*q = 0*.03 ^m-h^*q = 0*.03 ^l-h^*q < 0*.01	−0.14/−0.11/−0.14 ^l-m^*q = 0*.03 ^m-h^*q = 0*.03 ^l-h^*q = 0*.01	0.03/0.00/−0.06 ^l-m^*q = 0*.03 ^m-h^*q = 0*.06 ^l-h^*q = 0*.09	−0.09/−0.07/−0.06 ^l-m^*q = 0*.02 ^m-h^*q = 0*.01 ^l-h^*q = 0*.03	−0.25/−0.21/−0.17 ^l-m^*q = 0*.04 ^m-h^*q = 0*.03 ^l-h^*q = 0*.08	0.28/0.23/0.26 ^l-m^*q = 0*.06 ^m-h^*q = 0*.03 ^l-h^*q = 0*.02

(1) = General health, (2) = Mental health, (3) = Emotional role, (4) = Openness, (5) = Agreeableness, (6) = Adaptive coping strategies, (7) = Active maladaptive coping strategies, (8) = Non-active maladaptive coping strategies.

The value of Spearman’s Rho (rsp) is included for the groups of low, medium and high overstimulation (in this order), separated by slashes. The pairwise comparisons are included: l-m = low vs medium, l-h = low vs high, and m-h = medium vs high. The interpretation levels of Cohen’s q are as follows: (s) = small (q = 0.10–0.30), (m) = moderate (q = 0.31–0.50) and (l) = large (q= > 0.50).

The results showed that the differences in the correlations did not reach a perceptible effect size (*q* = ≤0.10), concluding that the level of aesthetic sensitivity did not present a moderator effect on the relationships, since the significant relationships between the variables were similar for the participants with low, medium and high aesthetic sensitivity.

Regarding the third objective, in order to know the configuration and relevance of each variable in the set of variables between all the variables and determine whether there are modifications due to the moderator effect of aesthetic sensitivity in the set of relationships obtained, a network analysis was carried out. The standardised weights of these relationships are presented in Table 5.

**Table 5:** Matrix of weights of the network of variables.

Variable	Network						
	(1)	(2)	(3)	(4)	(5)	(6)	(7)
Mental Health^(2)^	0.24						
Emotional Role^(3)^	0.14	0.44					
Openness^(4)^	0.01	−0.02	−0.14				
Agreeableness^(5)^	0.02	0.12	−0.03	0.17			
Adaptive Coping Strategies^(6)^	0.09	0.20	−0.04	0.24	0.10		
Active maladaptive coping strategies^(7)^	−0.06	−0.35	−0.07	0.04	0.01	0.10	
Non-active maladaptive coping strategies^(8)^	0.02	0.03	−0.04	0.02	−0.04	−0.18	0.22

(1) = General health, (2) = Mental health, (3) = Emotional role, (4) = Openness, (5) = Agreeableness, (6) = Adaptive coping strategies, (7) = Active maladaptive coping strategies, (8) = Non-active maladaptive coping strategies.

**Figure 1:**
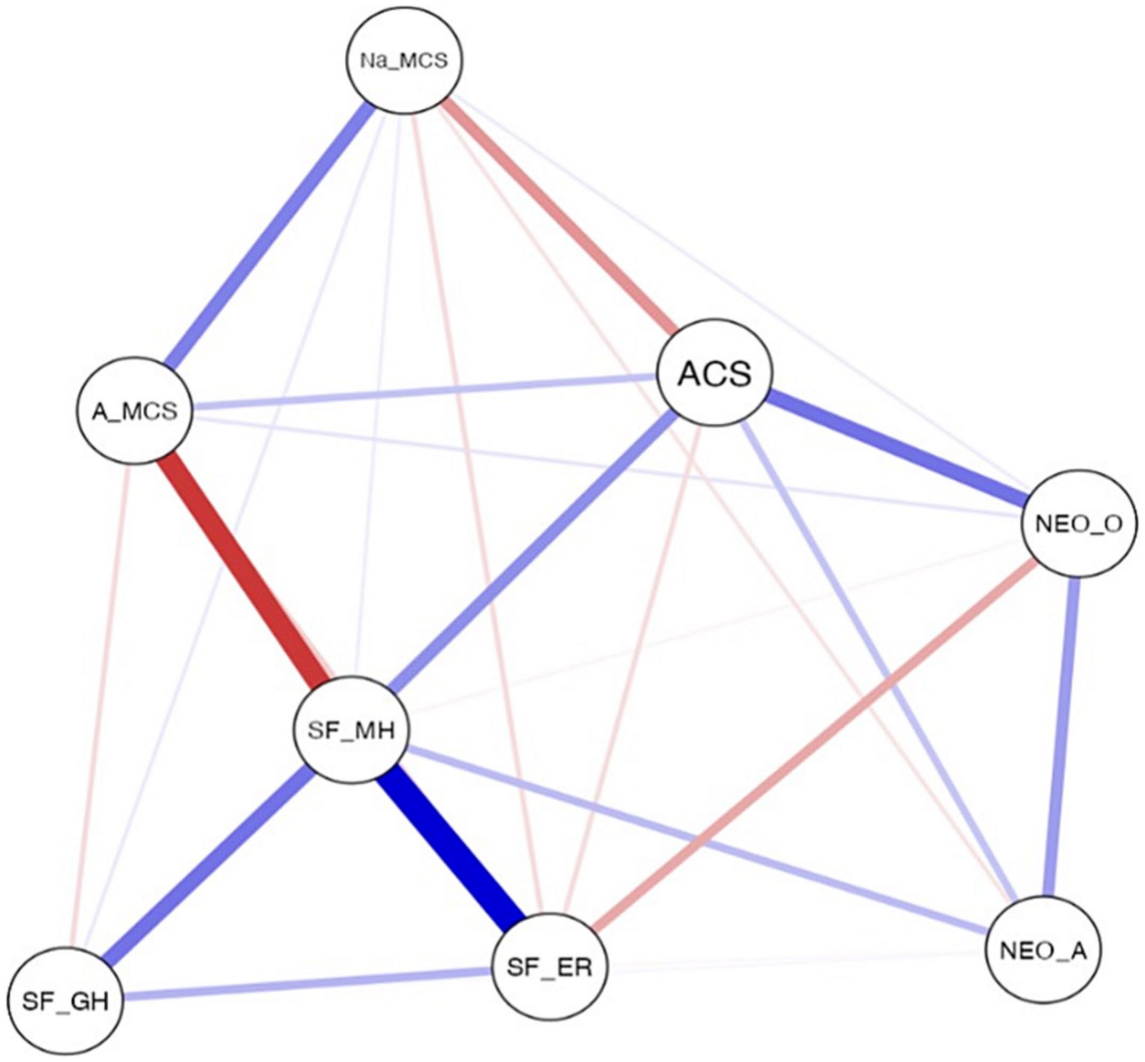
Network analysis of the variables of health-related quality of life, openness, agreeableness and coping strategies. SF_GM = General Health, SF_MH = Mental Health, SF_ER = Emotional role, NEO_O=Openness, NEO_A = Agreeableness, ACS = Adaptive coping strategies, A_MCS = Active maladaptive coping strategies, Na_MCS=Non-active maladaptive coping strategies.

Figure 1 shows that the three indicators of health-related quality of life were positively related to each other, with mental health presenting a stronger correlation with emotional role (*q* = 0.45) and general health (*q* = 0.24). Mental health appeared in a central position in the set of network variables, indicating the importance of this variable in the set. There was a positive correlation with adaptive coping strategies (*q* = 0.19) and a weak correlation with agreeableness (*q* = 0.10), as well as a negative correlation between mental health and active maladaptive coping strategies (*q* = −0.34).

Moreover, a negative and direct relationship was detected between emotional role and openness (*q* = −0.15), and a negative and indirect relationship was observed between agreeableness and openness (*q* = 0.17), with a positive relationship being identified between the two personality traits. However, these variables were not sufficiently intense to be in a central position in the set of the network, and thus they were in a more peripheral zone.

With regard to the coping strategies, a significant negative relationship was observed in non-active maladaptive coping strategies with adaptive coping strategies (*q =* −0.18) and a significant positive relationship was detected in non-active maladaptive coping strategies with active maladaptive coping strategies (*q* = 0.22). Adaptive coping strategies occupied a central position, since they were strongly related to other variables. The weakest positive relationship was obtained between adaptive coping strategies and active maladaptive coping strategies (*q* = 0.10).

Therefore, in the set of analysed variables, mental health and adaptive coping strategies presented the largest number of strong relationships with the rest of variables.

Secondly, with the aim of determining the existence of modifications due to the moderator effect of the level of aesthetic sensitivity in the set of relationships obtained, a network analysis was carried out. Table 6 shows these results as a function of the low, medium and high level of aesthetic sensitivity.

**Table 6:** Network analysis of the study variables as a function of aesthetic sensitivity level.

Variable	Aesthetic sensitivity level Low/Medium/High						
	(1)	(2)	(3)	(4)	(5)	(6)	(7)
Mental Health^(2)^	0.25/0.26/0.23						
Emotional Role^(3)^	0.15/0.10/0.14	0.43/0.41/0.41					
Openness^(4)^	<0.001/<0.001/<0.001	−0.01/<0.001/<0.001	−0.11/−0.10/−0.09				
Agreeableness^(5)^	0.03/0.01/<0.001	0.09/0.12/0.09	0.01/<0.001/<0.001	0.11/0.10/0.12			
Adaptive Coping Strategies^(6)^	0.09/0.06/0.07	0.17/0.14/0.18	−0.02/−0.01/−0.01	0.16/0.13/0.13	0.10/0.07/0.09		
Active maladaptive coping strategies^(7)^	−0.03/−0.07/−0.04	−0.32/−0.32/−0.34	−0.11/−0.09/−0.05	0.03/0.01/0.01	0.01/<0.001/0.01	0.10/0.03/0.02	
Non-active maladaptive coping strategies^(8)^	0.01/0.01/<0.001	0.01/<0.001/<0.001	0.01/−0.01/−0.03	0.03/0.01/0.01	−0.02/−0.01/−0.01	−0.21/−0.16/−0.11	0.22/0.17/0.19

(1) = General health, (2) = Mental health, (3) = Emotional role, (4) = Openness, (5) = Agreeableness, (6) = Adaptive coping strategies, (7) = Active maladaptive coping strategies, (8) = Non-active maladaptive coping strategies.

The standardised weights of the groups of low, medium and high aesthetic sensitivity are gathered (in this order) and separated by slashes.

**Figure 2:**
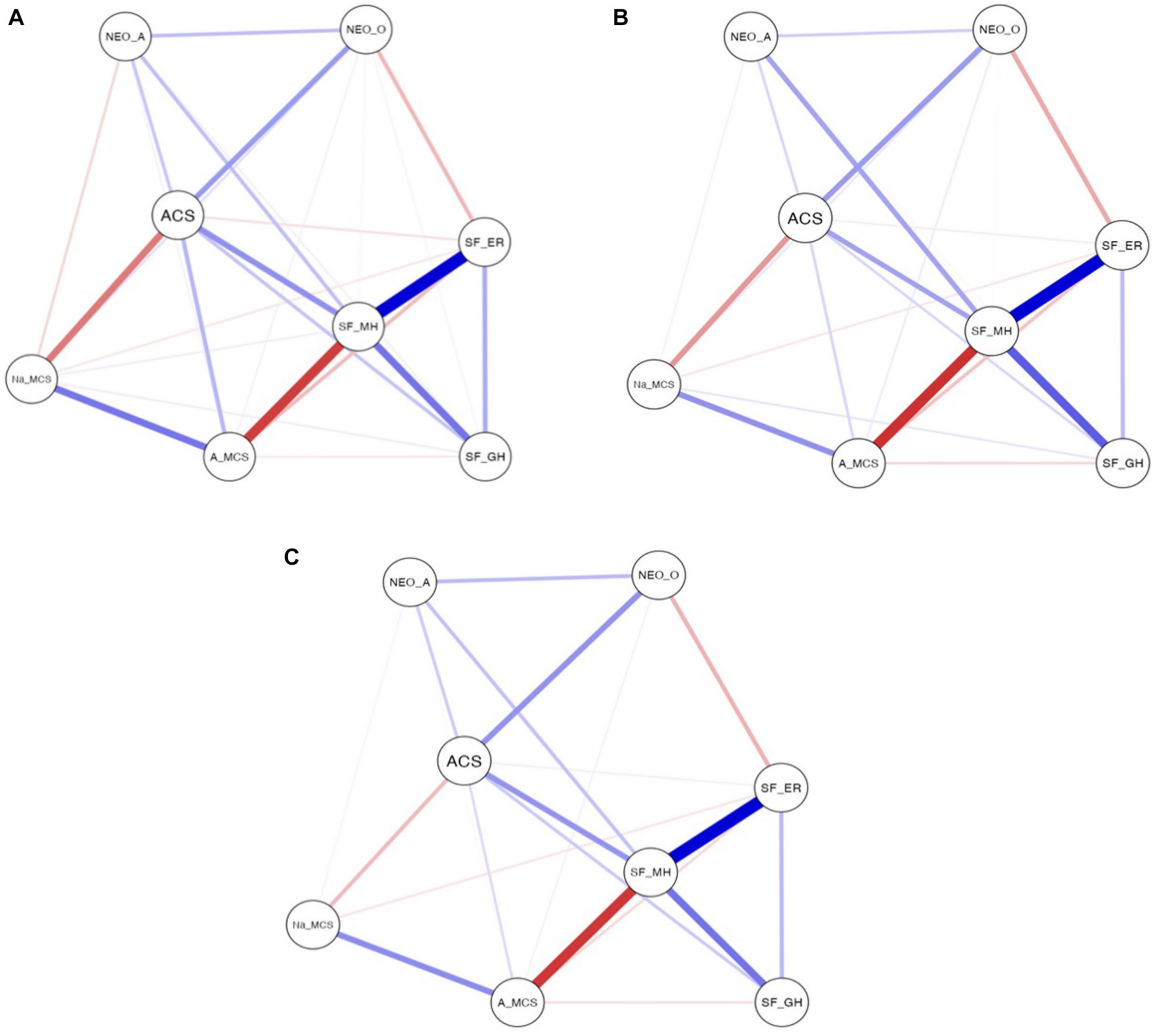
**(A–C)** Network analysis of the study variables with low, medium and high aesthetic sensitivity level. **(A)** Low aesthetic sensitivity level. **(B)** Medium aesthetic sensitivity level. **(C)** High aesthetic sensitivity level. SF_GM = General Health, SF_MH = Mental Health, SF_ER = Emotional role, NEO_O=Openness, NEO_A = Agreeableness, ACS = Adaptive coping strategies, A_MCS = Active maladaptive coping strategies, Na_MCS=Non-active maladaptive coping strategies.

Figure 2 shows that, despite the variation in the level of aesthetic sensitivity, the network generated by the relationships between the analysed variables was constant. In the low, medium and high levels of aesthetic sensitivity, the relationships were similar between the indicator of health-related quality of life mental health and the indicators emotional role and general health. Similarly, a positive correlation was obtained in all levels between mental health and adaptive coping strategies, and a weaker correlation between mental health and openness, as well as a negative relationship between mental health and active maladaptive coping strategies.

Therefore, the level of aesthetic sensitivity did not exert a moderator effect on the relationships between the indicators of health-related quality of life, the personality traits openness and agreeableness, and coping strategies.

## 4 Discussion

The general aim of this study was to determine the effect of aesthetic sensitivity as an essential facet or characteristic of highly sensitive people, reflecting the positive aspect of this personality trait. In a population with a representative number of people with SPS, we analysed health-related quality of life, openness to experience and agreeableness, and the coping strategies that these people use in their daily living.

The specific objectives were: (1) to explore the differences, as a function of the level of aesthetic sensitivity, in the indicators of health-related quality of life, openness and agreeableness, and coping strategies; (2) to analyse the relationships between pairs of variables and determine whether these relationships were moderated by the level of aesthetic sensitivity; and (3) to assess the relevance of each variable in the set of relationships between all variables and determine whether there are modifications due to the moderator effect of aesthetic sensitivity in the set of relationships obtained.

This study was carried out using network analysis, which is a model that allows integratively analysing network structures composed of different variables and understanding the influence of the variables from the groupings that are established, being able to occupy central positions (strongly connected) or peripheral positions (weakly connected) in the network. It represents a novel contribution to the state of the art regarding aesthetic sensitivity in people with high SPS.

In the general relationship between the variables, it was expected that the personality traits studied and the health-related quality of life indicators would occupy a central position and the coping strategies a peripheral position. Likewise, it was hypothesised that aesthetic sensitivity, especially high aesthetic sensitivity, could be exerting a moderating effect on variables that were not personality traits, specifically, indicators of health-related quality of life and coping strategies, producing a different grouping in the relationships between variables considered globally.

With respect to aesthetic sensitivity, the first contribution of this study demonstrates that high aesthetic sensitivity is accompanied by greater openness to experience and tendencies towards the social world, which are defined in agreeableness, altruism, trust, sympathy and/or forgiving or conciliatory attitudes. Furthermore, the participants with high aesthetic sensitivity tended to use adaptive coping strategies more frequently, although they showed a slightly worse functioning in the different areas of life, due to the way in which emotional reactivity to everyday problems affects the emotional state of people with SPS.

These findings are in agreement with those of previous studies that related this fact of SPS to the traits of openness and agreeableness (Smolewska et al., 2006; Trå et al., 2022). It is thus confirmed that, in addition to aesthetic sensitivity to the environment, people with SPS are also sensitive to the social world, with greater capacity to perceive and regulate emotions (Trå et al., 2022), creative fantasy (DeYoung, 2015) and empathy (Acevedo et al., 2017). Therefore, it is not surprising that people with high aesthetic sensitivity manage social situations more adequately, especially in extroverted people (Pérez-Chacón et al., 2023a,b).

From the network analysis, regarding the relationships established between pairs of variables, it is worth highlighting a positive relationship between the indicators of health-related quality of life, general health, mental health and emotional role. In other words, mental and physical health, as well as emotional well-being in the functioning of daily living, are associated with better health-related quality of life. Moreover, the personality traits openness and agreeableness and the use of adaptive coping strategies are associated with lower emotional distress. Although the cost in emotional distress associated with interpersonal situations is higher and correlates with health-related quality of life (Pérez-Chacón et al., 2023a,b), highly sensitive people can benefit from a better emotional and mental quality of life (Genizi et al., 2019) due to the attraction to the beauty of the arts and nature, which are strengths that, based on the theory of vantage sensitivity and positive psychology, contribute to emotional well-being.

These findings demonstrate the relevance of the traits openness and agreeableness in health-related quality of life, as well as the use of adaptive coping strategies. In this sense, in line with previous studies, high sensitivity is associated with openness (Smolewska et al., 2006; Lionetti et al., 2018) and agreeableness (Trå et al., 2022). Cognitive flexibility and emotional involvement, which define openness (McCrae and Costa, 1997; DeYoung, 2015), and empathy, cooperation and correct mind reading, which characterise agreeableness (Nettle and Liddle, 2008), are involved in the use of adaptive coping strategies (Connor-Smith and Flachsbart, 2007), such as emotional expression, social support, conflict resolution and cognitive restructuring.

Moreover, both openness and agreeableness are associated with better quality of life. Openness is related to emotional role and agreeableness is related to mental health, which is in line with the findings of Huang et al. (2017), who reported that openness and agreeableness are associated with better health-related quality of life in general. However, in this study, openness was positively related to mental health through adaptive coping strategies, and this relationship was stronger than the negative relationship of openness with emotional role, counteracting the distress related to functioning in daily living, which is explained from the theory of differential susceptibility (Belsky and Pluess, 2009; Pluess and Belsky, 2009), such as greater effects (positive or negative), that is, due to reactivity in favourable or unfavourable environments in highly sensitive people, as well as to the experience of negative internal states in response to the exposure to internal and external stimuli (Brindle et al., 2015).

In this line, previous studies show that the use of active maladaptive coping strategies (Connor-Smith and Flachsbart, 2007), such as the use of thoughts loaded with fantasy (DeYoung, 2015) that foster the desire that reality were different, are associated with lower emotional reactivity.

Lastly, with respect to the effect of aesthetic sensitivity on the analysed variables, the findings of this study indicate that the level of aesthetic sensitivity do not play a moderator role, with the organisation of the variables remaining constant depending on the relevance between them. Therefore, regardless of the level of aesthetic sensitivity, these people present openness to experience and agreeableness, experience good health-related quality of life, and frequently employ adaptive coping strategies. However, regarding the importance of the variables within the network, these findings are in disagreement with what was expected. Firstly, in the set of variables, the health-related quality of life indicators were the only variables with the greatest importance in the relationships, although personality traits showed a weaker relationship. Secondly, according to the level of aesthetic sensitivity, both in people with high aesthetic sensitivity and in those with medium and low aesthetic sensitivity, there were indicators of quality of life related to health, that is, mental health, physical health and emotional role or discomfort in everyday situations, which showed more importance. Thirdly, the relationships established at the three levels were identical, indicating that the studied qualities of the positive facet in people with SPS are similar, with only quantitative differences. These results could be explained by the relevance of functioning at a neuropsychological level in these people (Acevedo et al., 2017).

To sum up, aesthetic sensitivity in people with SPS is related to the personality traits of openness to experience and agreeableness. In turn, the use of adaptive coping strategies and mental health are associated with the personality traits, demonstrating that, in highly sensitive people, the presence of openness to experience and agreeableness contribute to minimising and counteracting reactivity in favourable or unfavourable environments. In this sense, it is important to point out that, in certain areas of functioning, such as the labour scope, strengths like empathy in healthcare and education professionals can become a risk factor for the development of compassion fatigue and burnout (Pérez-Chacón et al., 2021), being associated with the suffering experienced by these professionals in problematic situations of other people.

## 5 Conclusion

In conclusion, aesthetic sensitivity, which is a facet of SPS that perceives the aesthetics of the environment and socio-emotional aspects, is strongly related to characteristics of the personality traits openness and agreeableness. Moreover, these characteristics are associated with adaptive coping strategies, such as emotional expression and regulation, and with better management of problematic situations at work and other activities of daily living that produce emotional reactivity. Furthermore, mental health and adaptive coping strategies, associated with other relevant variables analysed in this study, acquire an important role in health-related quality of life. Lastly, no moderator effect was detected in aesthetic sensitivity on the mentioned relationships.

These results could be highly relevant and useful for the propositions of prevention and clinical intervention, as well as for counselling in the psychoeducational, labour and family scopes, amongst others. They contribute to underlining the importance of enhancing the strengths of people with SPS in playful and/or professional activities linked to the arts and nature or the animal world, as sources of emotional well-being and life satisfaction. In addition, from a therapeutic point of view, this is a way of compensating and managing the emotional distress inherent to this personality trait, which manifests with anxiety and depression, paying special attention to these issues when helping these people.

## 6 Limitations

Firstly, the use of a cross-sectional methodology is not compatible with drawing conclusions similar to those drawn from a longitudinal methodology. Secondly, the exclusive use of self-reported scales implies a risk of bias in the attribution and interpretation of the results. Lastly, the profession or type of labour activity of the participants was not considered as a study variable, which would have allowed determining possible differences and similarities between the participants of this study as a function of the field of knowledge (technical, law, science, arts..., etc.). Thus, it is convenient, from the associations of professionals and experts in high sensitivity, to pay attention to and delve into this facet, i.e., the positive side of SPS, in order to advance in the knowledge of the positive and negative variables that have an impact on the individual differences, as they influence emotional well-being and quality of life in different scopes, including health and the labour scope, amongst others.

Finally, it is important to mention those limitations that could have arisen from the online evaluation, such as the impossibility of controlling variables outside the study that could have interfered with the completion of the questionnaires. In future work, it would be advisable to expand data collection in person, in order to delve further into this topic and guarantee equal opportunities for the target population of the study.

## Data availability statement

The raw data supporting the conclusions of this article will be made available by the authors, without undue reservation.

## Ethics statement

Ethical approval was not required for the study involving humans in accordance with the local legislation and institutional requirements. Written informed consent to participate in this study was not required from the participants or the participants' legal guardians/next of kin in accordance with the national legislation and the institutional requirements.

## Author contributions

AC: Conceptualization, Data curation, Funding acquisition, Investigation, Resources, Writing – original draft, Writing – review & editing. MB-M: Conceptualization, Data curation, Investigation, Project administration, Supervision, Validation, Visualization, Writing – original draft, Writing – review & editing. FR: Data curation, Formal Analysis, Methodology, Software, Supervision, Validation, Writing – review & editing. MP-C: Conceptualization, Funding acquisition, Investigation, Resources, Writing – original draft, Writing – review & editing. MA-N: Conceptualization, Investigation, Project administration, Supervision, Validation, Visualization, Writing – original draft, Writing – review & editing.
